# Interaction between *Medicago truncatula* and *Pseudomonas fluorescens*: Evaluation of Costs and Benefits across an Elevated Atmospheric CO_2_

**DOI:** 10.1371/journal.pone.0045740

**Published:** 2012-09-21

**Authors:** Clémentine Lepinay, Thierry Rigaud, Christophe Salon, Philippe Lemanceau, Christophe Mougel

**Affiliations:** 1 INRA, UMR 1347 Agroécologie, Dijon, France; 2 CNRS, UMR CNRS/Université de Bourgogne 6282 Biogeosciences, Dijon, France; University of the West of England, United Kingdom

## Abstract

Soil microorganisms play a key role in both plants nutrition and health. Their relation with plant varies from mutualism to parasitism, according to the balance of costs and benefits for the two partners of the interaction. These interactions involved the liberation of plant organic compounds via rhizodeposition. Modification of atmospheric CO_2_ concentration may affect rhizodeposition and as a consequence trophic interactions that bind plants and microorganisms. Positive effect of elevated CO_2_ on plants are rather well known but consequences for micoorganisms and their interactions with plants are still poorly understood. A gnotobiotic system has been developed to study the interaction between *Medicago truncatula* Jemalong J5 and the mutualistic bacteria *Pseudomonas fluorescens* strain C7R12 under two atmospheric CO_2_ concentrations: ambient (365 ppm) versus enriched (750 ppm). Costs and benefits for each partner have been determined over time by measuring plant development and growth, the C and N contents of the various plant parts and the density of the bacteria in rhizosphere compartments. Following the increase in CO_2_, there was a beneficial effect of *P. fluorescens* C7R12 on development, vegetative growth, and C/N content of *M. truncatula*. Concerning plant reproduction, an early seed production was noticed in presence of the bacterial strain combined with increased atmospheric CO_2_ conditions. Paradoxically, this transient increase in seed production was correlated with a decrease in bacterial density in the rhizosphere soil, revealing a cost of increased CO_2_ for the bacterial strain. This shift of costs-benefits ratio disappeared later during the plant growth. In conclusion, the increase in CO_2_ concentration modifies transiently the cost-benefit balance in favor of the plant. These results may be explained either by a competition between the two partners or a change in bacterial physiology. The ecosystem functioning depends on the stability of many plant-microbe associations that abiotic factors can disrupt.

## Introduction

The interactions between plants and soil microorganisms are mainly based on trophic relationships [1]. In mutualistic interactions, the plant supplies to microbes carbon compounds, which drive both development of microbial population and their activities [2], [3]. In exchange, microbes provide the plant with inorganic and/or organic compounds necessary to its growth [4]. The compounds are costly to produce for each partner but these costs are balanced by the benefits. This subtle cost-benefit balance results from a co-evolution between the two organisms [5]–[8]. However, the present stability of such interactions often masks a conflict of interest between the two partners. It has been shown that some associations may be mutualistic or antagonistic according to variations in the proximate environment, biotic or abiotic, of the partners (e.g. [9]). How modulation of environmental factors can affect the nature of the association (mutualistic or parasitic) remains overlooked in the case of interactions between plants and soil microorganisms. Some studies showed that changes in atmospheric carbon dioxide (CO_2_) induce differential competition for other elements between plants and microorganisms [10]–[13], but a potential shift in the balance of costs-benefits between the partners of the association has never been investigated.

Plant-microbe association in the rhizosphere (the area of soil around roots which is strongly influenced by the metabolism of the plant) is hugely dependent on the plant photosynthetic rate, which determines the amount of rhizodeposits potentially used by microorganisms [14]. The photosynthetic rate is itself determined by abiotic factors, such as availability of soil water and soil mineral elements [15] mainly nitrogen [16], and above all the atmospheric CO_2_. Modulating the plant carbon budget through a variation of the atmospheric CO_2_ concentration is a valuable approach to go further in the understanding of plant-microbe interactions in the rhizosphere. The effects of elevated atmospheric CO_2_ on plants are rather well known, increasing their overall fitness [16]–[18]. However, the responses of microbial component to elevated CO_2_ are still poorly understood. Some studies highlighted changes in physiology and composition of microbial communities [19] or in a bigger bacterial density because of the increase in the carrying capacity of the rhizosphere environment due to the larger amount of rhizodeposits [20]. Up to now, the feedback effect on the plant has never been investigated.

**Table 1:** Results of the ANOVA with repeated measures explaining the number of leaves of *Medicago truncatula*, as a function of inoculation conditions (inoculated or not with *Pseudomonas fluorescens* C7R12), CO_2_ concentration (ambient, 365 ppm or enriched, 750 ppm), the plant developmental stage (V and Fp, see text) and theexperimental replicate.

Source of variation	dfNum	dfDenom	F	P
**Between subjects**				
Replicate	1	69	113.06	**<0.0001**
Inoculation	1	69	11.20	**0.0013**
CO_2_	1	69	38.62	**<0.0001**
Inoculation*replicate	1	69	0.72	0.40
CO_2_*replicate	1	69	0.48	0.49
CO_2_*inoculation	1	69	12.85	**0.0006**
CO_2_*inoculation*replicate	1	69	0.02	0.90
**Within subjects**				
Time	6	64	3891.13	**<0.0001**
Time*Replicate	6	64	51.93	**<0.0001**
Time*Inoculation	6	64	3.59	**0.0040**
Time*CO_2_	6	64	25.70	**<0.0001**
Time*Inoculation*replicate	6	64	0.87	0.52
Time*CO_2_*replicate	6	64	8.33	**<0.0001**
Time*CO_2_*inoculation	6	64	2.65	**0.0235**
Time*CO_2_*inoculation*replicate	6	64	1.56	0.17

*Medicago truncatula* is a model plant for legumes [21]–[24], mainly because of its symbiotic associations with rhizobia [4], [25], its large synteny with several legumes [26], its small genome size [27] and the availability of genetic and genomic resources. Moreover, *M. truncatula* interacts with various telluric microbes including plant growth promoting rhizobacteria (PGPR) such as *Pseudomonas fluorescens* [28]. Among them, *P. fluorescens* strain C7R12, a mutant resistant to rifampicin [29] of a strain isolated from a soil naturally suppressive to a major soilborne disease [30], was shown to promote growth and mycorrhization of *M. truncatula* [31], and to efficiently colonize the rhizosphere and the root tissues of this plant species [32], [33]. Here, the interaction between *M. truncatula* and the mutualist bacteria, *P. fluorescens* C7R12, was studied using a gnotobiotic system specifically developed for this purpose. In order to investigate the sole effect of *P. fluorescens*, *M. truncatula* was grown in gnotobiotic conditions in the absence of resident symbionts. More precisely, the dynamic of costs and benefits for *M. truncatula* and the *P. fluorescens* C7R12 was characterized during their interaction from vegetative to reproductive stages and this work allowed to answer the following questions: (i) How bacterial presence impacts *a priori* the positive effect of elevated CO_2_ concentrations on plant trait?; (ii)Due to an increased carbon availability, does this effect lead to alterations in bacterial density ? and (iii) do we observe any change in the mutualistic association?

**Table 2:** Linear models explaining the vegetative traits of *Medicago truncatula* as a function of inoculation conditions (inoculated or not with *Pseudomonas fluorescens* C7R12), CO_2_ concentration (ambient, 365 ppm or enriched, 750 ppm), the plant developmental stage (V, Fp and Pf, see text) and the experimental replicate (nested factor), excepting for the C/N ratios (C,D) which were measured in the second experiment only for the Fp and Pf stages.

Source of variation	df	Sum of Squares	F Ratio	P
**(A) Shoot dry biomass**				
Replicate	1	0.44	7.91	**0.0061**
Inoculation [replicate]	2	0.33	2.94	0.06
**CO_2_ [replicate]**	2	3.96	35.25	**<0.0001**
**Developmental stage [replicate]**	4	49.54	220.69	**<0.0001**
Inoculation*CO_2_ [replicate]	2	0.12	1.05	0.35
Inoculation*Developmental stage [replicate]	4	0.78	3.47	**0.0111**
CO_2_*Developmental stage [replicate]	4	0.26	1.14	0.34
Inoculation*CO_2_*Developmental stage [replicate]	4	0.11	0.48	0.75
Error	88	4.94		
**(B) Root dry biomass**				
Replicate	1	0.00	0.05	0.83
**Inoculation [replicate]**	2	0.61	5.48	**0.0057**
**CO_2_ [replicate]**	2	0.90	8.07	**0.0006**
**Developmental stage [replicate]**	4	23.21	104.40	**<0.0001**
Inoculation*CO_2_ [replicate]	2	0.04	0.35	0.71
Inoculation*Developmental stage [replicate]	4	0.28	1.27	0.29
CO_2_*Developmental stage [replicate]	4	0.34	1.52	0.20
Inoculation*CO_2_*Developmental stage [replicate]	4	0.17	0.75	0.56
Error	88	4.89		
**(C) C/N of shoot**				
Inoculation	1	0.01	3.81	0.06
**CO_2_**	1	0.49	204.12	**<0.0001**
**Developmental stage**	1	0.67	279.58	**<0.0001**
Inoculation*CO_2_	1	0.01	2.97	0.09
Inoculation*Developmental stage	1	0.09	35.54	**<0.0001**
CO_2_*Developmental stage	1	0.00	1.47	0.23
Inoculation*CO_2_*Developmental stage	1	0.00	0.72	0.40
Error	75	1.35		
**(D) C/N of root**				
Inoculation	1	0.00	3.95	0.05
**CO_2_**	1	0.01	12.04	**0.0009**
**Developmental stage**	1	0.06	125.76	**<0.0001**
Inoculation*CO_2_	1	0.01	22.84	**<0.0001**
Inoculation*Developmental stage	1	0.01	13.01	**0.0006**
CO_2_*Developmental stage	1	0.00	4.24	**0.0433**
Inoculation*CO_2_*Developmental stage	1	0.00	0.01	0.92
Error	75	0.13		

## Materials and Methods

### Experimental Design

Two experimental replicates were performed successively at different times in the same controlled conditions. The two experiments were carried out with two interacting treatments: bacteria (substrate being inoculated or not with bacteria) and CO_2_ concentration (plant grown under ambient (365 ppm) or elevated (750 ppm) atmospheric CO_2_ concentration). Five replicates were used for each treatment combination. Under each condition, plant development was characterized three times during the growth cycle: during the vegetative period (V, 29 days post-sowing), during flowering, at the beginning of pods maturation (Fp, 43 days post-sowing) and during a later stage of reproduction, where some flowers were still present but most of pods were maturing on plants (Pf, 63 days post-sowing). This resulted in 2 experimental replicates×4 treatments (inoculated *versus* no-inoculated and ambient *versus* elevated CO_2_)×3 stages investigated (V, Fp and Pf)×5 replicates (pots), equalling 120 pots in total.

### Medicago Truncatula Growth Conditions

Seeds of *M. truncatula* Gaertn. cv. Jemalong line J5 were selected for homogeneous weight (between 4.5 and 5 mg) in order to homogenize germination. Seeds were scarified, surface sterilized, pre-germinated [34] and vernalized by a passage at 4°C for 10 days to accelerate the beginning of flowering [35]. Before being sown, seeds were placed at 20°C for 24 h in the dark.

Seeds were sown in stainless steel pots containing a substrate composed of attapulgite and expanded clay mixed in proportion 1/1 (v/v). The pots were specifically developed in order to allow plant growth and maintain gnotobiotic conditions (see Figure S1). They were sterilized together with substrate using gamma rays (internal dose of 45 kGy) (IONISOS, Meylan, France) before seeds were sown. All the pots were watered with 50 ml of nutrient solution at 10 milliequivalents of nitrogen (mM N) and composed of filtered (0.2 µm) stock solutions with the following ion composition: KNO_3_ (2.02×10^−3^ mol l^−1^), K_2_HPO_4_ (7.92×10^−4^ mol l^−1^), Ca(NO)_2_+4H_2_O (3×10^−3^ mol l^−1^), MgSO_4_ (1.2×10^−3^ mol l^−1^), NaNO_3_ (2.25×10^−3^ mol l^−1^), H_3_Bo_3_ (1.92×10^−8^ mol l^−1^), MnSo_4_+7H_2_O (1.06×10^−8^ mol l^−1^), ZnSO_4_+7H_2_O (7×10^−10^ mol l^−1^), CuSo_4_ (3.2×10^−9^ mol l^−1^), Na_2_MoO_4_+2H_2_O (1×10^−9^ mol l^−1^), CoCl_2_+6H_2_O (8.4×10^−11^ mol l^−1^) and C_14_H_18_FeN_3_Na_2_O_10_ (5×10^−2^ mol l^−1^). For each atmospheric CO_2_ condition, half of the pots were inoculated with the bacterium *P. fluorescens* C7R12.

**Table 3:** Linear models explaining the reproductive traits of *Medicago truncatula* as a function of inoculation conditions (inoculated or not with *Pseudomonas fluorescens* C7R12), CO_2_ concentration (ambient, 365 ppm or enriched, 750 ppm) and the plant developmental stage (Fp and Pf, see text), excepting for the pod mass (C) which was measured in the second experimental replicate only.

Source of variation	df	Sum of Squares	F Ratio	P
**(A) Number of flowers**				
Replicate	1	6.83	202.81	**<0.0001**
Inoculation [replicate]	2	0.17	2.55	0.09
**CO_2_ [replicate]**	2	2.72	40.43	**<0.0001**
**Developmental stage [replicate]**	2	2.14	31.73	**<0.0001**
Inoculation*CO_2_ [replicate]	2	0.06	0.82	0.45
Inoculation*Developmental stage [replicate]	2	0.21	3.07	0.05
CO_2_*Developmental stage [replicate]	2	0.63	9.36	**0.0003**
Inoculation*CO_2_*Developmental stage [replicate]	2	0.14	2.04	0.14
Error	60	2.02		
**(B) Number of pods**				
Replicate	1	0.53	8.09	**0.0061**
Inoculation [replicate]	2	0.09	0.67	0.52
**CO_2_ [replicate]**	2	2.49	19.03	**<0.0001**
**Developmental stage [replicate]**	2	0.97	7.39	**0.0014**
Inoculation*CO_2_ [replicate]	2	0.26	2.02	0.14
Inoculation*Developmental stage [replicate]	2	0.14	1.05	0.36
CO_2_*Developmental stage [replicate]	2	4.72	36.05	**<0.0001**
Inoculation*CO_2_*Developmental stage [replicate]	2	0.17	1.29	0.28
Error	60	3.93		
**(C) Mass of one pod**				
Inoculation	1	0.03	1.33	0.26
**CO_2_**	1	0.49	26.05	**<0.0001**
**Developmental stage**	1	5.81	306.18	**<0.0001**
Inoculation*CO_2_	1	0.06	2.96	0.10
Inoculation*Developmental stage	1	0.07	3.80	0.06
CO_2_*Developmental stage	1	0.00	0.25	0.62
Inoculation*CO_2_*Developmental stage	1	0.01	0.69	0.41
Error	37	6.92		

A glass pot attached to the cover allowed the plant development to occur in a sterile environment at the beginning of its growth. After 15 days of seedling growth, glass pots were removed and gas-proof silicone (RHODORSIL® RTV 3325 with 5% of catalyst; GACHES CHIMIE Spécialités, Illzach, France) was disposed around the hypocotyl to maintain gnotobiotic conditions in the soil while allowing aerial parts of the plant to grow at atmospheric conditions chosen.

Plants were cultivated for 63 days in growth chamber with a 16/8 h light/dark photoperiod and 23/18°C light/dark thermoperiod. The amount of photosynthetic active radiation at pots height was 500 µEinsteins m^−2^ s^−1^. The growth chamber had a capacity of about 9 m^3^ and could be used or not as a gas-proof system to allow the enrichment of the total atmosphere of the growth chamber. Such only one gas-proof growth chamber was available, two successive experiment were undertaken for each experiment, 30 pots at ambient CO_2_ concentration (measured at 365 ppm) in a no gas-proof version (15 inoculated and 15 not inoculated) following by 30 pots at 750 ppm in a gasproof version (15 inoculated and 15 not inoculated). To obtain a constant and uniformly enriched atmosphere, the CO_2_ concentration was measured continuously during experiment using an infrared gas analyser (IRGA; PP system; Ciras, Montigny le Bretonneux, France) and maintained by automatic CO_2_ injection as in Voisin *et al*. [36].

**Table 4:** ANOVA explaining the density of *Pseudomonas fluorescens* C7R12 in the rhizosphere soil (A) and the plant roots (B), as a function of CO_2_ concentration (ambient, 365 ppm or enriched, 750 ppm), the plant developmental stage (V, Fp and Pf, see text) and the experimental replicate (nested factor).

Source of variation	df	Sum ofSquares	F Ratio	P
**(A) Bacterial density in** **Rhizosphere soil**				
Replicate	1	0.93	11.06	**0.0017**
CO_2_ [replicate]	2	0.62	3.66	**0.0334**
Developmental stage [replicate]	4	3.74	11.12	**<0.0001**
CO_2_*Developmental stage [replicate]	4	2.11	6.28	**0.0004**
Error	46	3.87		
**(B) Bacterial density in Root**				
Replicate	1	5.23	30.95	**<0.0001**
CO_2_ [replicate]	2	2.04	6.04	**0.0047**
Developmental stage [replicate]	4	14.89	22.01	**<0.0001**
CO_2_*Developmental stage [replicate]	4	2.37	3.50	**0.0141**
Error	46	7.78		

### Preparation of *Pseudomonas fluorescens* Strain C7R12 Inoculums

*Pseudomonas fluorescens* C7R12 was grown on King B agar medium plates (KMB agar) [37] for 48 h at 25°C and cells were harvested and washed once in sterile demineralized water. The cell density was determined spectrophotometrically at 600 nm and adjusted with sterile demineralized water to inoculate the substrate at 10^6^ CFU (Colony Forming Unit) g^−1^ of dry substrate. The same volume of demineralized water was added to the uninoculated treatment to obtain the same substrate humidity.

### Quantification of Plant Traits

The kinetics of plant development was followed by counting the number of leaves twice a week. Reproductive potential was estimated as the number of pods and seeds at the Fp and Pf stages.

**Figure 1:**
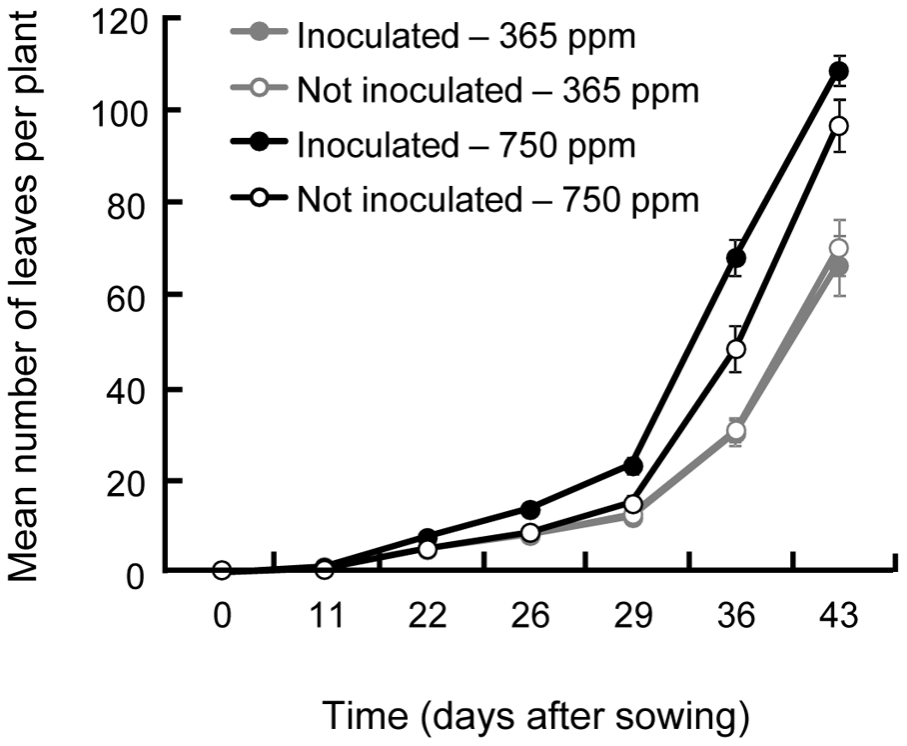
Average number of leaves of *Medicago truncatula* over its growth. The number of leaves depends on the CO_2_ concentration and the inoculation with *Pseudomonas fluorescens* C7R12. An ANOVA for repeated measures was made because this trait was measured several times on the same plants. Standard errors are represented by vertical bars. V and Fp corresponds to the plant developmental stages analysed in the experiment.

The shoot and root biomass increased over time and were significantly higher when CO_2_ concentration increased (76.52% and 41.30% respectively; Table 2A, B). A positive *P. fluorescens* C7R12 effect was found only at stage V for shoot (Table 2A) where biomass was twice higher with the bacteria. In the root compartment, this positive effect of *P. fluorescens* C7R12 inoculation was found regardless the other parameters (Table 2B). For these vegetative plant traits, the effect of *P. fluorescens* C7R12 and CO_2_ never interact (Table 2A, B).

**Figure 2:**
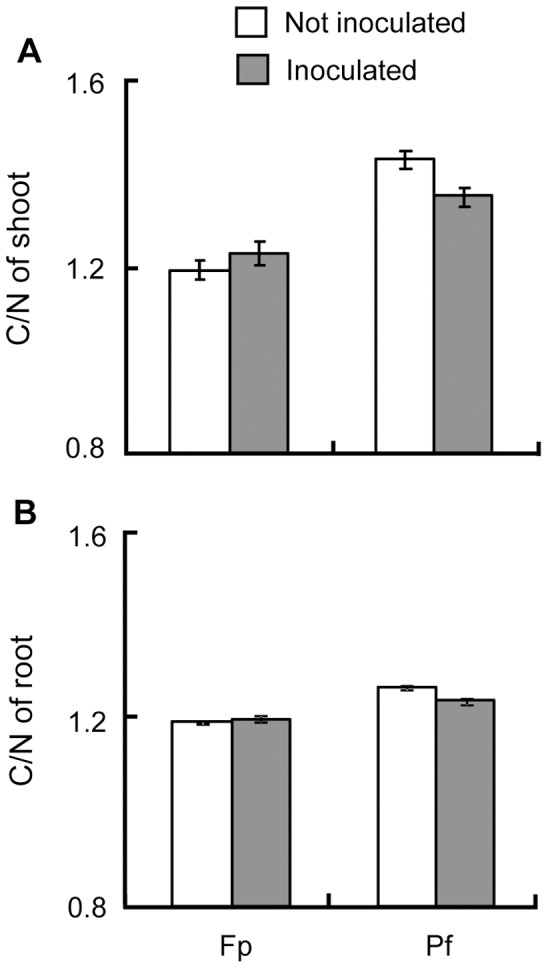
Average C/N ratio for shoot and root of *Medicago truncatula*. Average C/N ratio for the shoot (A) and the root (B) depends on the developmental stage (Fp, Pf, see text) and the condition of inoculation (Inoculated or not with *Pseudomonas fluorescens* C7R12). Data were log-transformed to achieve normality and equal variances respectively tested by Shapiro-Wilk and Bartlett tests. Carbon-Nitrogen ratio, measured during the second experiment replicate only, was analysed by linear model with condition of inoculation, CO_2_ concentration and developmental stage as fixed factors and with their interactions. Standard errors are represented by vertical bars.

**Figure 3:**
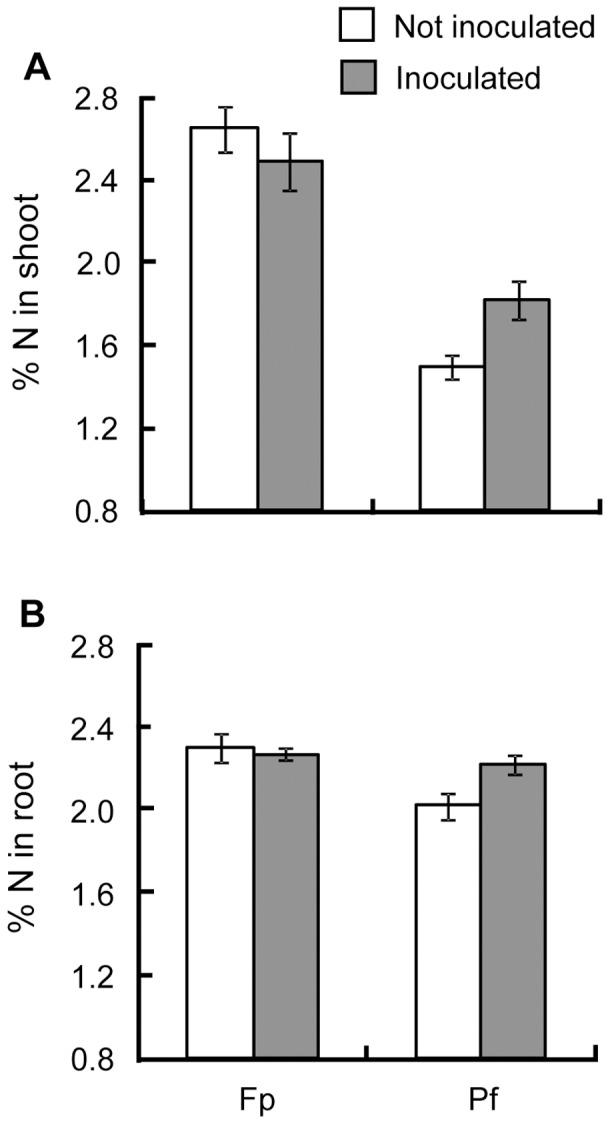
Average percentage of Nitrogen in *Medicago truncatula* shoot and root. Average percentage of Nitrogen in the shoot (A) and the root (B) depends on the developmental stage (Fp, Pf, see text) and the condition of inoculation (Inoculated or not with *Pseudomonas fluorescens* C7R12). The percentage of N, measured during the second experiment replicate only, was analysed by linear model with condition of inoculation, CO_2_ concentration and developmental stage as fixed factors and with their interactions. Standard errors are represented by vertical bars.

In a qualitative viewpoint, the shoot and root C/N ratio raised over time. Shoot C/N ratio was more important under increased CO_2_ concentration, regardless the date of analysis (Table 2C, D). For root, the C/N ratio was higher at stage Fp for 750 ppm (Table 2D) but CO_2_ concentration played no role at stage Pf. The condition of inoculation played a role in interaction with the developmental stage for the two compartments. The C/N ratio increased with the developmental stage but was lower at stage Pf when the bacteria interact with the plant (Figure 2A, B). These effects at Pf stage on the C/N ratio may be linked to the N content in those compartments at this stage (Figure 3A, B).

At each growth stage, five plants per condition were analysed as follows. Shoots, roots, pods and seeds were separated and their biomass was determined after desiccation at 105°C for 24 h. Dry shoots, roots, pods and seeds were ground in a grinding bowl with a steel ball in a mixer-mill (vibro-broyeur Retsch MM 301). The carbon (C) and nitrogen (N) contents in the different plant compartments were quantified using Dumas procedure in an elemental analyser (ThermoElectron NC2500 elemental analyser).

### Quantification of *Pseudomonas fluorescens* C7R12 Density and Gnotobiotic Conditions Assessment

The density of *P. fluorescens* C7R12 in both the rhizosphere soil and the root was determined during the interaction with the plant by counting the number of CFU on KMB agar supplemented with rifampicin (100 mg l^−1^). For each date of analysis, the initial suspension of soil adhering to the roots (rhizosphere soil) was sampled by vortexing the root in 100 ml of sterile demineralized water. Once cleared from their rhizosphere soil, roots were dried on sterile filter paper and 1.5 g taken randomly at different locations of the roots were ground in a sterile mortar. The ground roots obtained were recovered in 10 ml of sterile demineralized water to form initial root suspensions. Bacterial population of this root suspensions correspond to bacteria attached to the root surface (rhizoplane) and present in the roots (endophyte).

**Figure 4:**
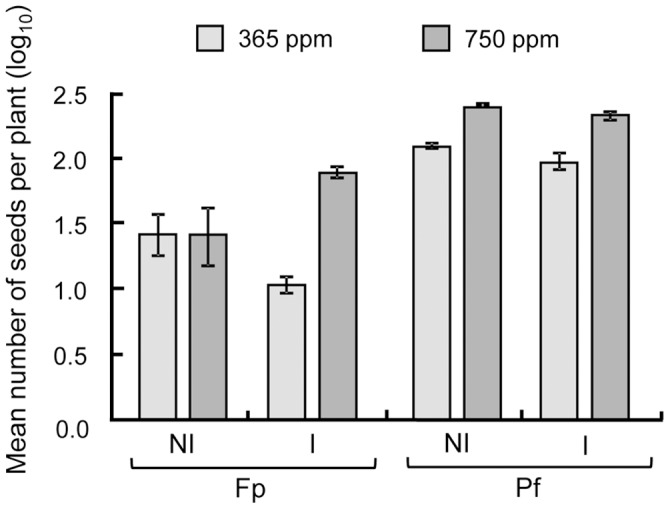
Average number of seeds per plant. Average number of seeds per plant depends on the CO_2_ concentration (ambient, 365 ppm or enriched, 750 ppm), the condition of inoculation (I: inoculated or NI: not with *Pseudomonas fluorescens* C7R12) and the developmental stage of *Medicago truncatula* (Fp, Pf, see text). Data were log-transformed to achieve normality and equal variances respectively tested by Shapiro-Wilk and Bartlett tests. Number of seeds, measured during the second experiment replicate only, was analysed by linear model with condition of inoculation, CO_2_ concentration and developmental stage as fixed factors and with their interactions. Standard errors are represented by vertical bars.

Potential bacteria and fungi contaminants were analysed. For bacteria, the medium TSA 1/10 (trypcase soy agar) was used. This medium was composed, for 1 l of demineralised water, of 15 g tryptone, 5 g soy peptone, 5 g sodium chloride and 15 g agar. The pH was adjusted to 7.3. The medium was sterilized at 110°C for 45 min and cycloheximide was added (100 mg l^−1^). For fungi, the malt acid medium was used. It was constituted, for 1 l of demineralized water, of 10 g of malt and 15 g agar, then autoclaved for 20 min at 120°C. Before use, 250 mg l^−1^ of citric acid, streptomycin (100 mg l^−1^) and chlortetracycline (50 mg l^−1^) were added.

**Figure 5:**
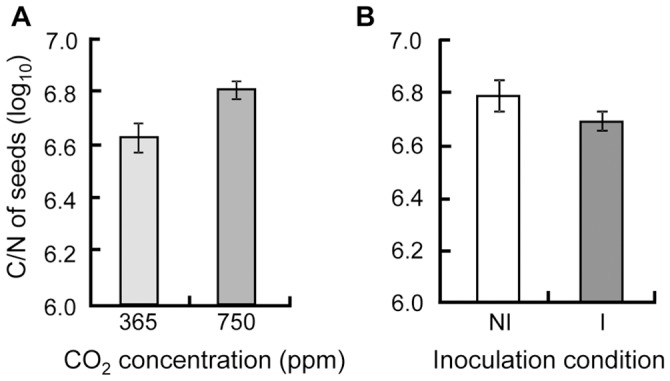
Carbon-Nitrogen ratio of Medicago truncatula seeds. Carbon-Nitrogen ratio of seeds depends on (A) the CO_2_ concentration (ambient, 365 ppm or enriched, 750 ppm) and (B) the condition of inoculation (I: inoculated or NI: not with *Pseudomonas fluorescens* C7R12). Data were log-transformed to achieve normality and equal variances respectively tested by Shapiro-Wilk and Bartlett tests. Carbon-Nitrogen ratio, measured during the second experiment replicate only, was analysed by linear model with condition of inoculation, CO_2_ concentration and developmental stage as fixed factors and with their interactions. Standard errors are represented by vertical bars.

### Statistical Analysis

Normality and equal variances were tested respectively by Shapiro-Wilk and Bartlett tests. When necessary, data were log-transformed to achieve normality. Most plant data were analysed by linear models with condition of inoculation, CO_2_ concentration and developmental stage as fixed factors and with their interactions, all being nested within the experiment replicate factor. Since parameters on seeds were only measured during the second experiment replicate, this replicate factor was not taken into account in the analysis. For the leaves number, an ANOVA for repeated measures was made because this trait was measured several times on the same plants. The densities of microorganisms were analysed with ANOVA using as explanatory fixed factors the developmental stage and CO_2_ concentration and their interactions, nested within the replicate experiment factor.

All values presented in the text are mean ± s.e.m. The analyses were performed using the statistical software JMP 5.0.1. with an alpha level of 0.05.

**Table 5:** Linear models explaining the quantitative (A) and qualitative (B,C,D) aspects of seeds of *Medicago truncatula* as a function of inoculation conditions (inoculated or not with *Pseudomonas fluorescens* C7R12), CO_2_ concentration (ambient, 365 ppm or enriched, 750 ppm) and the plant developmental stage (Fp and Pf, see text).

Source of variation	df	Sum of Squares	F Ratio	P
**(A) Number of seeds per plant**				
Inoculation	1	0.01	0.42	0.52
**CO_2_**	1	0.56	25.82	**<0.0001**
**Developmental stage**	1	4.42	202.25	**<0.0001**
Inoculation*CO_2_	1	0.63	28.81	**<0.0001**
Inoculation*Developmental stage	1	0.05	2.18	0.15
CO_2_*Developmental stage	1	0.02	0.93	0.34
Inoculation*CO_2_*Developmental stage	1	0.34	15.53	**0.0006**
Error	25	0.55		
**(B) Mass of one seed**				
Inoculation	1	0.11	1.46	0.24
CO_2_	1	0.05	0.72	0.40
**Developmental stage**	1	5.28	72.20	**<0.0001**
Inoculation*CO_2_	1	0.02	0.33	0.57
Inoculation*Developmental stage	1	0.06	0.89	0.36
CO_2_*Developmental stage	1	0.02	0.22	0.64
Inoculation*CO_2_*Developmental stage	1	0.01	0.12	0.73
Error	32	7.26		
**(C) C/N Ratio in seeds**				
**Inoculation**	1	0.27	6.17	**0.0162**
**CO_2_**	1	0.19	4.33	**0.0423**
**Developmental stage**	1	0.62	14.38	**0.0004**
Inoculation*CO_2_	1	0.00	0.00	0.99
Inoculation*Developmental stage	1	0.00	0.01	0.93
CO_2_*Developmental stage	1	0.00	0.02	0.88
Inoculation*CO_2_*Developmental stage	1	0.09	2.07	0.16
Error	52	2.25		
**(D) %N in seeds**				
**Inoculation**	1	0.37	5.91	**0.0186**
**CO_2_**	1	0.29	4.67	**0.0353**
**Developmental stage**	1	5.41	87.30	**<0.0001**
Inoculation*CO_2_	1	0.04	0.67	0.42
Inoculation*Developmental stage	1	0.05	0.75	0.39
CO_2_*Developmental stage	1	0.06	1.00	0.32
Inoculation*CO_2_*Developmental stage	1	0.06	1.04	0.31
Error	59	11.01		

## Results

No bacterial or fungal contaminant was detected, at thresholds detections, respectively 23.35 and 233.53 CFU g^−1^ of dry substrate, during the experiment. For most of the traits measured on the plant or bacteria, there was a variation between the two experimental replicates (Tables 1, 2, 3 and 4). This variation nevertheless never masked other sources of variation, notably interactions between factors, results being congruent between replicates. This source of variation will therefore not be discussed further.

**Figure 6:**
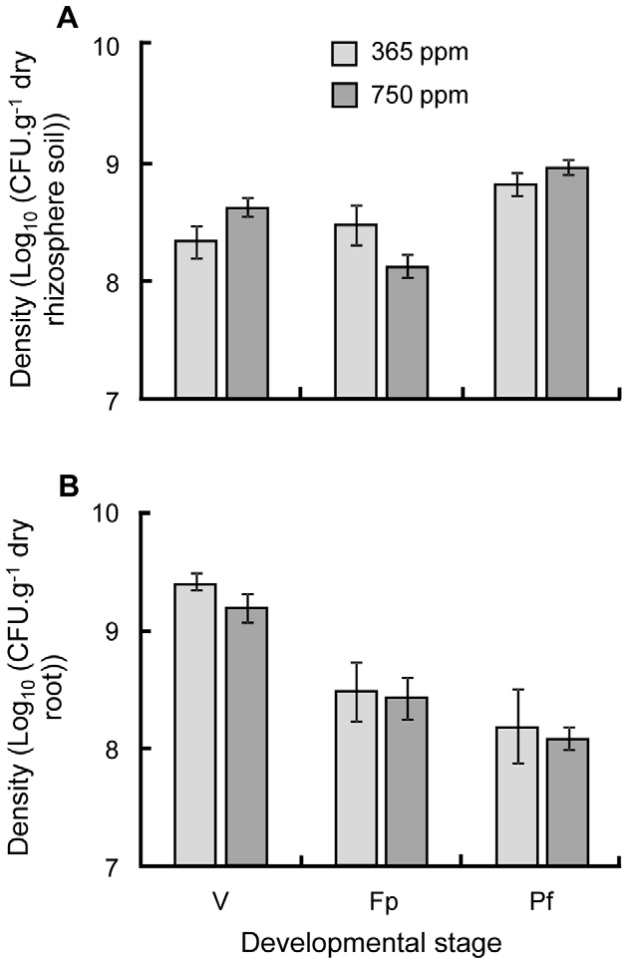
Average density of *Pseudomonas fluorescens* C7R12 over time. The density of the bacterial strain was measured for the developmental stages chosen (V: vegetative; Fp: Flowers, some pods; Pf: Pods, some flowers) in compartments (A) rhizosphere soil and (B) root. Data were log-transformed to achieve normality and equal variances respectively tested by Shapiro-Wilk and Bartlett tests. The densities of microorganisms were analysed with ANOVA using as explanatory fixed factors the developmental stage and CO_2_ concentration and their interactions, nested within the replicate experiment factor. Standard errors are represented by vertical bars.

### CO_2_ and *P. fluorescens* C7R12 Effect on Vegetative Growth and Development of *M. truncatula*

The number of leaves increased exponentially with time, reflecting plant development and growth. The leaves appeared more rapidly at elevated CO_2_ concentration. This CO_2_-induced acceleration of development was amplified when plants were inoculated with *P. fluorescens* C7R12. (Table 1, Figure 1).

### CO_2_ and *P. fluorescens* C7R12 Effect on Reproductive Traits of *M. truncatula*

For the first experimental replicate, the flowers appeared after 34 days and the pods after 35 days, in the ambient CO_2_ concentration. In contrast, when plants were subjected to an enrichment of the CO_2_ concentration, flowering began at 28 days and the pod apparition at 32 days.

The number of flowers and pods were both significantly influenced by CO_2_ concentration in interaction with the developmental stage (Table 3A, B). Plants growing at high CO_2_ concentration had more flowers at stage Pf (ambient: 12.15±2.72 versus elevated: 16.47±2.09) and 3.5 times more pods at this stage. The presence of *P. fluorescens* C7R12 never influenced these two traits significantly, either alone or in interaction with another factor (Table 3A, B).

Total number of seeds per plant rose over time (Table 5A, Figure 4), and the condition of inoculation influenced this dynamics in interaction with CO_2_ concentration. At stage Fp, the plants interacting with bacteria produced 7.45 times more seeds at high CO_2_ concentration, while plants without bacteria produced the same number of seeds whatever the CO_2_ concentration. At stage Pf, this CO_2_-induced increase in the number of seeds was still observable. However, at this stage, the number of seeds was not significantly influenced by the inoculation (Figure 5).

The pod and seed masses showed a rise through developmental stages (Tables 3C and 4B) and the pod mass was higher under elevated CO_2_ (Table 3C).

Finally, the C/N ratio of seeds (Table 5C) was higher when the plant received elevated CO_2_ concentration (Figure 4A). This ratio was lower in the presence of the bacterial strain (Figure 4B) and over developmental stages (Table 5C, data not shown) but no interaction between these factors was significant (Table 5C). This was due to a higher N content in seeds with the bacterial strain (Table 5D).

### CO_2_ Effect on *P. fluorescens* C7R12 Fitness

Preliminary tests confirmed the absence of difference in *P. fluorescens* C7R12 densities between the two CO_2_ treatments after 15 days of growth (F_1,19_ = 0.21, P = 0.65) and thus validate the initial conditions of our experiment.

The bacterial density in the rhizosphere soil compartment was influenced by CO_2_ concentration in interaction with the plant developmental stage (Table 4A). At ambient CO_2_ concentration, the bacterial density was more or less constant whereas at enriched CO_2_ there was a drop in bacterial density during the stage Fp (Figure 6A). There was a general decrease in concentration of bacteria associated intimately with root (including bacteria attached to the root surface (rhizoplane) and present in the roots (endophyte)) over developmental stages (Table 4B), and, again, the effect of CO_2_ concentration led to a bacterial density slightly lower under high CO_2_ concentration (Figure 6B).

## Discussion

### CO_2_ Enrichment and *P. fluorescens* C7R12 Interact Positively on *M. truncatula* Development and Growth

Carbon dioxide enrichment in the atmosphere usually leads to a faster shoot development and positive effects on both shoot and root growth. These effects have been often demonstrated in the literature [38], [39]. They presumably result from an increase in the plant photosynthetic rate due to the higher CO_2_ enrichment, the enhanced photosynthates production being invested in plant biomass [16], [18].

During the early periods of the growth cycle, the presence of the bacterium improved shoot growth. During this vegetative stage, plants release numerous carbon compounds [34], made available for bacteria, which promotes in turn root and shoot growth. As observed for shoot biomass at vegetative stage, that of roots was also increased when the *P. fluorescens* and *M. truncatula* interacted regardless to the developmental stage or the CO_2_ concentration. This result confirms the effect of *P. fluorescens* C7R12 on root growth and architecture on *M. truncatula* [31], [33]. Moreover, when the plant interacts with the bacteria, its N content in shoot and root parts was higher regardless the CO_2_ concentration revealing a positive effect of *P. fluorescens* C7R12 on nitrogen nutrition of *M. truncatula*. If some N_2_-fixing *P. fluorescens* strains have been identified [40], there is no evidence that *P. fluorescens* C7R12 has this capacity. Another hypothesis is linked to the ability of *P. fluorescens* C7R12 to increase plant iron nutrition [41] which allows better photosynthetic functioning [42] and so an efficient intake of CO_2_ and probably of nutrients necessary for this process.

### Interaction between CO_2_ and *P. fluorescens* C7R12 on Reproductive Traits of *M. truncatula*

The increase in both plant biomass and the number of reproductive structures observed in our study throughout the growth cycle demonstrates that our specifically developed gnotobiotic system did not perturb plant development and growth.

Under elevated CO_2_, the higher biomass production impacted positively on the number of reproductive organs, flowers and pods. These observations are consistent with other studies [43], [44] and can be explained by physiological changes when the plant receives a larger amount of carbon.

The dynamics in seeds production differed according to CO_2_ conditions and the plant developmental stages during the interaction between the plant and the bacteria. In presence of *P. fluorescens* C7R12, the plant produced a high number of seeds at the early reproductive stage under high CO_2_. Later in the development, the amount of seeds produced was similar with and without the bacteria under elevated CO_2_. Reaching faster (beginning of flowering appeared 6 days before) a high reproductive capacity is likely to give a selective advantage to the plant in natural environments. Early production of numerous offspring could provide a competitive advantage for future germination, and/or compensate early plant mortality in an unpredictable environment [45]. This phenomenon could also be interpreted in another way. It is well known that living organisms can plastically invest in early reproduction when they experience a stress such as parasitic pressure (e.g. [46], [47]). Here, such a phenomenon could appear paradoxical because it is observed in conditions *a priori* favourable for the plant, i.e. under high CO_2_ concentration and in presence of a mutualistic partner. However, the transient decrease in bacterial density in the soil at this stage of plant development (see discussion below) could induce a transient stress for the plant, which could plastically adjust its reproductive effort at that stage. In addition to this increased early reproductive effort, seeds contained more nitrogen when the plant interacted with *P. fluorescens* C7R12, an additional advantage because nitrogen is fundamental in seeds development and in its quality [48]. This fits the prediction that the inoculation with *P. fluorescens* C7R12 provides an advantage in terms of plant reproduction under elevated CO_2_ concentration.

### CO_2_ and *M. truncatula* Effect on *P. fluorescens* C7R12

The hypothesis was that plants subjected to a high CO_2_ concentration, release more carbon compounds, would support a higher bacterial density in the rhizosphere corresponding to an increase in its carrying capacity [13]. However, our experimental results did not support this hypothesis. Globally, bacteria did not increase in density and even had transiently a lower fitness during the plant development. At Fp stage, there was a decrease in bacterial density in the rhizosphere soil under higher CO_2_ condition. This transient drop in bacterial density may be due to a tradeoff between fueling soil micro-organisms and ensuring the reproductive potential. As such, the decrease in density at Fp stage could therefore coincide with both quantitative and qualitative changes [34] of carbon compounds released by plants in the rhizosphere [49]. Another explanation would be that competition occurred between the two partners: the seed C/N ratio was lower in the presence of bacteria which may result from a lower N allocation to the bacterium [16]. Therefore, interpreting the impact of an increased quantity of carbon made available for *P. fluorescens* C7R12 fitness is not straightforward.

### Changes in the Cost/Benefit Balance Due to Increase in Atmospheric CO_2_

*Pseudomonas fluorescens* C7R12 often provides an advantage in plant growth, during the vegetative stages, independent of CO_2_ conditions [50]. Although this growth advantage is interesting in an agricultural context for yield enhancement, it is however more questionable for the plant from an evolutionary point of view if it is not translated in an advantage in producing more viable offspring. Bacteria provided a qualitative advantage to the plant fitness, seeds containing more nitrogen. This advantage was associated with the presence of the bacteria, whatever the CO_2_ conditions, which only marginally influenced the bacterial density in the final stage of our study. The above advantage is therefore not linked to bacterial quantities.

In contrast with this qualitative advantage provided by the bacteria, one quantitative advantage was CO_2_ condition dependent. Inoculated plants produced more seeds than uninoculated ones at an early stage of reproduction, and under high concentration of CO_2_ only. In this later case, however, it is worth noting that this transient increase in plant reproductive capacity corresponded with the transient drop in bacterial density under high CO_2_ condition. Paradoxically, the benefit for *M. truncatula* to interact with *P. fluorescens* C7R12 was linked with a decrease in bacterial density. This could be due to an amplification or modification of bacterial metabolic activity [13], [51], and the benefit for the plant could result from a quantitative and/or qualitative change of the plant metabolites supplied by the bacterium [13], [52], [53]. However, it could also mean that the plant could transiently benefit from the decrease in bacterial density. Under such a hypothesis, interacting with too manybacteria at the stage where reproduction begins would be more detrimental than beneficial for the plant fitness. This would mean that the nature of the interaction changed with the CO_2_ increase. In that case, the plants would be the driver of changes in quantity and/or quality of their metabolites leading to decreased bacterial density. Finzi *et al*. [52] have shown that under elevated atmospheric CO_2_ condition, rhizodeposits are enriched in carbon while depleted in nitrogen. Consequently these rhizodeposits could be more difficult to metabolize for microorganisms lacking nitrogen [19]. Even if such a hypothesis remains to be tested, the mutualistic relationship between *M. truncatula* and *P. fluorescens* C7R12 therefore seems to be transiently destabilized under elevated CO_2_, revealing that under nearly unlimited carbon, supplying the mutualistic bacteria could be more costly than beneficial for the plant.

## Supporting Information

Figure S1 **Description and illustration of a gnotobiotic pot.** Gnotobiotic pot (A) made for the experiment allowing the growth of *Medicago truncatula* in microbiologically controlled conditions because of a glass pot (1) fixed to the cover with a plate (2) during 15 days at the beginning of growth. After this date, glass pot was removed and silicone was disposed around the hypocotyl (B). The plant was watered through a 0.2 µm filter (3). Air circulation is done through two air filters (4,5). The water outlet was controlled manually with a clamp (6). Figures (C) and (D) show more advanced developmental stage, corresponding to 35 days without and with the strain respectively, in CO_2_ ambient conditions.
(DOCX)
