# Supplementary material for: Interaction between Medicago truncatula and Pseudomonas fluorescens: Evaluation of Costs and Benefits across an Elevated Atmospheric CO2
Source: PLoS One. 2012 Sep 21;7(9):e45740. doi: 10.1371/journal.pone.0045740 (PMC3448688; doi:10.1371/journal.pone.0045740)
Supplement: Figure S1 — Description and illustration of a gnotobiotic pot. Gnotobiotic pot (A) made for the experiment allowing the growth of Medicago truncatula in microbiologically controlled conditions because of a glass pot (1) fixed to the cover with a plate (2) during 15 days at the beginning of growth. After this date, glass pot was removed and silicone was disposed around the hypocotyl (B). The plant was watered through a 0.2 µm filter (3). Air circulation is done through two air filters (4,5). The water outlet was controlled manually with a clamp (6). Figures (C) and (D) show more advanced developmental stage, corresponding to 35 days without and with the strain respectively, in CO2 ambient conditions. (DOCX) [file pone.0045740.s001.docx]

Supporting information – Figure S1
